# Teaching pain recognition through art: the Ramsay-Caravaggio sedation scale

**DOI:** 10.1186/s13052-018-0453-5

**Published:** 2018-01-31

**Authors:** Federico Poropat, Giorgio Cozzi, Andrea Magnolato, Lorenzo Monasta, Fabio Borrometi, Baruch Krauss, Alessandro Ventura, Egidio Barbi

**Affiliations:** 1 Institute for Maternal and Child Health - IRCCS “Burlo Garofolo” – Trieste, Via dell’Istria 65/1, 34137 Trieste, Italy; 2 University of Trieste, Trieste, Italy; 3 Pediatric Pain Service and Palliative Care, Department of Oncology, Pausilipon Hospital, AORN Santobono Pausilipon, Naples, Italy; 4 Division of Emergency Medicine, Boston Children’s Hospital, Boston, Massachusetts USA; 5 Department of Pediatrics, Harvard Medical School, Boston, Massachusetts USA

**Keywords:** Clinical observation is a key component of medical skills, Visual skills are hard to teach in the formal lessons, Ramsay sedation scale is a clinical score to measure patient’s depth of sedation during procedures, Adding visual art masterpieces to the standard lessons doesn’t improve the clinical visual skills but enhances the interest on the topic presented

## Abstract

**Background:**

Clinical observation is a key component of medical ability, enabling immediate evaluation of the patient’s emotional state and contributing to a clinical clue that leads to final decision making. In medical schools, the art of learning to look can be taught using medical humanities and especially visual arts. By presenting a Ramsay sedation score (RSS) integrated with Caravaggio’s paintings during a procedural sedation conference for pediatric residents, we want to test the effectiveness of this approach to improve the quality of learning.

**Methods:**

In this preliminary study, we presented videos showing sedated pediatric patients in the setting of a procedural sedation lesson to two randomized groups of residents, one attending a lesson on RSS explained through the masterpieces of Caravaggio, the other without artistic support. A week later we tested their learning with ten multi-choice questions focused on theoretical questions about sedation monitoring and ten more questions focused on recognizing the appropriate RSS viewing the videos. The primary outcome was the comparison of the total number of RSS layers properly recognized in both groups. We also evaluated the appreciation of the residents of the use of works of art integrated with the lesson.

**Results:**

Eleven students were randomized to each group. Two residents in the standard lesson did not attend the test. The percentage of correct answers on the theoretical part was similar, 82% in the art group and 89% in the other (*p* > 0.05). No difference was found in the video recognition part of the RSS recognition test. Residents exposed to paintings shown great appreciation for the integration of the lesson with the Caravaggio’s masterpieces.

**Conclusions:**

Adding artwork to a standard medical conference does not improve the performance of student tests, although this approach has been greatly appreciated by residents.

**Electronic supplementary material:**

The online version of this article (10.1186/s13052-018-0453-5) contains supplementary material, which is available to authorized users.

## Background

Clinical observation is a key component of medical skills. This is particularly true in the field of Pediatrics, in which the first clinical impression, even antecedent physical examination, may guide most of the decision-making. Observational and communicative skills are of essential importance in the measurement and management of anxiety, fear, and pain in children.

Teaching these skill is of paramount importance.

For centuries we have learned through visual arts. Since ancient times, with the majority of people being illiterate, paintings and sculptures, through their symbolism, were frequently commissioned and used to divulge stories and explain laws and rules. In this way, visual arts have always represented an effective educative tool.

Great artists have the power to convey immediate feelings and impressions that would have been transformed in teachings and enduring memories. Michelangelo Merisi (September 29, 1571 – July 18, 1610), universally known as Caravaggio, from the name of his hometown, is one of them. With the unique features of his work, he deeply renewed the art of painting influencing all artists of his time and beyond.

Procedural sedation is the standard of care for painful procedures in children and, in this setting, monitoring patients’ consciousness and response to painful stimuli plays a major role in defining the depth of sedation and in targeting appropriate drugs’ administration. The Ramsay sedation scale is one the most used clinical instruments to monitor the depth of sedation of patients in this setting [1].

Some studies show that exposure to work of arts by mean of repeated guided visits with an art expert to a museum can increase student’ skills [2, 3].

To the best of our knowledge, no study tested the impact of commented art images in a teaching lecture setting.

With the aim of improving the quality of our lectures during residency, we developed a modified Ramsay sedation scale adding details of Caravaggio’s paintings, in order to visually explain the scale. We tested the impact of this approach in a dedicated lecture in two groups of residents of the post-graduate medical school of pediatrics.

## Methods

We conducted this preliminary study at the tertiary level, university teaching, children’s hospital, Institute for Maternal and Child Health IRCCS Burlo Garofolo, Trieste, Italy, in the course of February 2017.

The study was approved by the Ethical Review Board. It was performed during the lectures organized for the training of pediatric residents in procedural sedation outside the operating room. Usually, the aim of the first lecture of 50 min is to explain basic principles of sedation and monitoring. In this setting the Ramsay sedation scale is a widely used instrument, which measures patient’s depth of sedation during procedures [1]. It consists of six depth levels of sedation, from awake and anxious (level 1) to unresponsive to any stimuli (level 6).

To carry out this study, a pediatric Anesthesiologist and two other Pediatricians trained in procedural sedation, after a meeting with an art historian, chose the details of Caravaggio’s paintings that, in their opinion, were most suggestive for each level of the Ramsay scale and they developed what we defined as the Ramsay-Caravaggio sedation scale (Additional file 1).

Before starting the lecture we asked to all residents attending the training if they have already known the Ramsay scale. Residents who have already known the scale were excluded from the study. Thereafter, residents were randomized in two groups of students.

The first group attended the lecture using Caravaggio’s paintings to explain the different levels of sedation considered in the Ramsay scale. The second group attended an analogous lecture, with the same teacher, but without any art image support.

One week after the lecture the residents of both groups tested their knowledge about Ramsay scale.

The evaluation test consisted of ten multiple-choice questions focusing on the theoretical issues of monitoring in procedural sedation, while other ten questions focused on the recognition of the appropriate level of the Ramsay scale, watching ten different videos, showing children during procedural sedation.

The residents who underwent the lecture in which the Ramsay scale was explained with details of Caravaggio’s paintings filled out an anonymous questionnaire about their opinion about the use of art in this setting.

The primary outcome of the study was the total number of Ramsay sedation levels correctly associated to each video in both groups. Secondarily, we tested the appreciation’s grade of residents about the use of art during frontal lectures.

### Statistical analysis

To evaluate the difference in the proportions of correct answers between the two groups we carried out two-tailed Fisher exact tests. We then calculated the number of correct answers given by each student and, to evaluate the difference in the performance on all six level of the Ramsay scale we compared the medians and interquartile ranges and carried out a Mann-Whitney rank-sum test.

## Results

In this study, 25 residents of Pediatrics underwent a frontal lecture about the principle of sedation and the Ramsay scale. Three students were excluded because they declared that they have already known the Ramsay scale. Therefore, 22 students were randomized, 11 to attend the lecture with the use of Caravaggio’s paintings to explain each level of the Ramsay scale and 11 to attend the standard lecture. Two students in the standard lecture group did not attend the test.

The percentage of correct multiple choice questions in the theoretical part was respectively 82% and 81% in the standard group (*p* > 0,05).

We found slight differences in the proportion of correct answers given during the test on the Ramsay scale. Actually, students in the art group had a not significantly worse performance on five out of six scales, while on the fourth item, the students of the art group performed significantly better, with 100% correct answers vs. 56% of the controls group. Even considering the median number of correct answer between the two groups, we found no difference, with both groups at 3 correct answers (Table 1).

**Table 1:** Correct answers to test on Ramsay sedation scale, and median number of correct answers: frequencies and percentages or median and interquartile range

Correct answers	Control Group, n. 9	Caravaggio Group, n. 11	*p*-value*	Total, n. 20
Ramsay 1	2 (22%)	2 (18%)	1.000	4 (20%)
Ramsay 2	0 (0%)	1 (9%)	1.000	1 (5%)
Ramsay 3	4 (44%)	2 (18%)	0.336	6 (30%)
Ramsay 4	5 (56%)	11 (100%)	0.026	16 (80%)
Ramsay 5	9 (100%)	11 (100%)	–	20 (100%)
Ramsay 6	8 (89%)	6 (55%)	0.157	14 (70%)
Total	3 (3 – 4)	3 (3 – 3)	0.575	3 (3 – 3)

*p-values refers to two-tailed Fisher exact test for comparison between proportions, and to Mann-Whitney rank-sum test to comparison between medians

The answers from the questionnaire showed a significant appreciation by the students exposed to the works of art (Table 2).

**Table 2:** Evaluation of the residents’ apreciation for the integration of the lesson with the Caravaggio’s masterpieces

Questions	Frequencies (percentages)
1. What do you think about the use of painting artworks as teaching tools in medical classes?	
- A useful tool	11 (100%)
- A potential distraction from key messages	0
- A waste of time	0
- None of above	0
2. In your opinion, can the evaluation of pain, anxiety and fear through a painting modify your ability of clinical interpretation ability?	
- Yes, it can be useful	9 (82%)
- No, it is too far from real life	0
- No, art is usually too cryptic to be easily understood	2 (18%)
- None of above	0
3. With regards to the Ramsay scale class you attended, in your opinion the paintings were:	
- Appropriate for the score represented	8 (73%)
- Not appropriate for the score represented	3 (27%)
- Wrong and misleading for the score represented	0
- None of above	0
4. With regards to the Ramsay scale class you attended, do you consider the images of the paintings:	
- Helpful for detecting the scores in the video	11 (100%)
- Not helpful for detecting the scores in the video	0
- Misleading	0
- None of above	0
5. Regarding the Ramsay scale class you attended, is there a particular image you have been impressed by:	
- Yes (specified the image)	8 (73%)
- Yes but I don’t remember which one	2 (18%)
- No	1 (9%)
- I don’t know	0
6. In your opinion, the evaluation of a piece of art with a didactic purpose makes sense …	
- … even if is illustrated by a doctor	9 (82%)
- … only if illustrated by an expert	1 (9%)
- … only if seen in real life	1 (9%)
- I don’t know	0

All the students answered that painting artworks were a useful tool. Nine students (82%) considered a piece of art as a useful device to figure out pain, anxiety, and fear in clinical evaluation. Two (18%) answered that art is usually too cryptic to be easily understood. The paintings were considered appropriate for the RSS by 8 students (73%) and all students considered the painting’s images helpful for detecting the Ramsay scores in the video. Participants reported to be impressed by a particular image: 2 (18%) were impressed by “*Penitent Magadalene”* (corresponding to RSS 3), 4 (36%) by “*Giuditta e Oloferne”* (corresponding to RSS 5), 2 (18%) by “*Conversione di San Paolo”* (corresponding to RSS 4). One student was not impressed by any picture. At last, 8 students (82%) answered that a work of art can be useful in the learning process even if a doctor illustrates it.

## Discussion

This study shows that adding visual art elements in the setting of a frontal medical lesson does not improve residents’ knowledge on a specific issue measured in terms of a written test and recognition of appropriate levels of sedation through videos. Even though residents showed a great appreciation for the integration of the lesson with the paintings.

Using visual arts has been reported to improve observational skills, increasing the ability to notice and to better assess symptoms, such as color, size, shape of a skin lesion [4, 5]. Moreover visual arts can open our minds to doubt, to consider other possible interpretations, seeing beyond the first view promoting strategies to analyze, compare and decipher visual clues [6]. Visual arts also enhances our capacity to capture facial expressions and emotions, improving communication skills, listening and analytic thinking, and so leading to the development of a visual expertise, defined as the ability to extract from an image the maximum of observations, information, insights, and meanings [5, 7, 8].

We arbitrarily chose to use Caravaggio’s masterpieces because of the peculiar features of his painting. Thanks to a completely new use of lights and shadows, and a realistic representation of bodies with all their natural traits and imperfections, Caravaggio’s painting technique portrayed humans no more as idealized, static and unemotional figures, but as real people. His painting represented a revolution for that time and a break point with the mannerism of Renaissance to him contemporary. Caravaggio portrayed his subjects as much as possible similar to their real appearance. Most of the times he chose to portray plebeians, like shop boys or prostitutes, in their natural pose and expressions, during their daily activities, using reality as the only source of inspiration, far away from the idealized world pursued by Renaissance artworks. In his paintings, a poorly defined background, with black as the predominant color, and a revolutionary use of light increase definition of bodies’ volumes and emphasize the atmosphere and feelings of the portrayed scene. His troubled life, scarred by a murder, which forced him to escape throughout Italy until his final amnesty, contributed to influence his art and to create a myth around this “damned” painter. Thanks to Caravaggio, paintings were no more a place where reality can be re-organized according to the beauty and perfection of human soul, but a place in which reality strikes us with its dramatic force and expressivity.

Visual Thinking Strategies (VTS) define a specific evidence-based approach to teaching, focused on the mental strategies people use to find meanings in a piece of art, aimed to enhance critical thinking and language skills [8]. The use of VTS in medical education has proven to increase students’ observational and relational skills [6]. Moreover, studies have shown that art helps to preserve the humanistic aspects of medical science. Students who have enriched their medical training with the study of art are more prone to see in front of them a “person”, and therefore have enhanced listening skills that allow a better doctor-patient relationship [6].

While evidence shows that exposure to work of arts by mean of repeated guided visits with an art expert to a museum can increase student’ skills, this approach requires many hours, specific tutorial skills and expertise and a dedicated setting [8]. On the other hand, to our knowledge, our study was the first that investigated how adding visual art elements during a lecture influence observational skills of students and there are no previous reported examples of teaching an instrument of clinical evaluation with the use of art. We found that the simple exposure to dedicated and commented art works in the setting of a medical lecture did not have a measurable impact on students test performance, but it was very appreciated.

This study has some limitations. First, the lesson was performed by a pediatric anesthesiologist with a limited knowledge on art and painting, who had received a previous simple informal training by an art historian by mean of a discussion of the paintings and of Caravaggio poetry. Second, the exposure to paintings was brief, embedded in a lesson limited at a single 50 min power point presentation, which is far less than the substantial exposure to works of art, such as visiting a museum for a set of hours with an art historian, reported by Katz and others [3, 6]. Third, the number of residents involved was limited and we checked students’ skills only one week after the lecture, so we did not investigate if the students who assisted the lecture with visual arts elements could have a more enduring memory of the scale.

## Conclusion

Our study shows that adding art pieces to a standard medical lecture may not improve students’ test performance, even if this approach was highly appreciated by residents. Further studies with a greater sample size are needed to confirm our findings and to investigate other possible application of similar teaching techniques in medical education.

## Additional file

Additional file 1: Ramsay-Caravaggio sedation scale. (PDF 1446 kb)

## Abbreviations

RSS: Ramsay sedation scale
VTS: Visual Thinking Strategies
