# Supplementary material for: Teaching pain recognition through art: the Ramsay-Caravaggio sedation scale
Source: Ital J Pediatr. 2018 Jan 31;44:20. doi: 10.1186/s13052-018-0453-5 (PMC5793413; doi:10.1186/s13052-018-0453-5)
Supplement: Additional file 1: — Ramsay-Caravaggio sedation scale. (PDF 1446 kb) [file 13052_2018_453_MOESM1_ESM.pdf]

Depth of sedation	Ramsay definition	Caravaggio paintings (details)	Paint description
1	Awake and anxious, agitated or restless	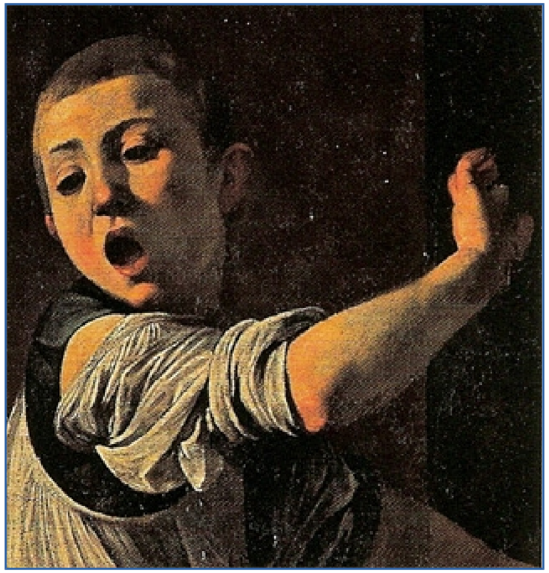	*Martirio di San Matteo (detail), Cappella Contarelli, Church of San Luigi dei Francesi, Rome.* The painting depicts the martyrdom of St Matthew, assassinated at the end of a mass during which he opposed the marriage of the brother ot the deceased king of Ethiopia and his niece, Iphigenia, who had previously consecrated her virginity to God. The detail of the painting portrays a scared believer who turn away from the scene. The face is taut, his eyes look down avoiding the spectator’s gaze, the mouth is opened in awe. The body rotated and the raised arm betrays the intention to flee

2	Awake and cooperative, oriented and calm	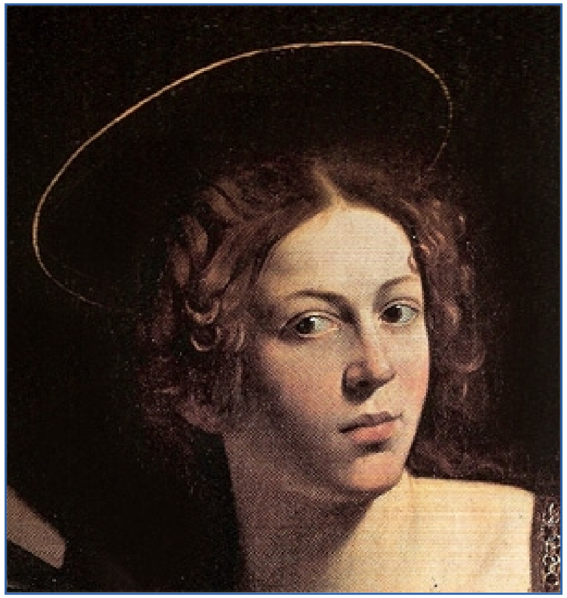	*Santa Caterina d’Alessandria (detail), Thyssen-Bornemisza Museum, Madrid*<br>The painting depicts saint Catherine of Alexandria surrounded by instruments of torture (the toothed wheel and the sword), with which she was murdered having refused to repudiate the Christian faith. Despite the Saint is surrounded by death tools, the face seems serene, the facial expression does not reveal emotions of discomfort or fear. The eyes stare at the observer with attentive and interrogative look, her lips are not contracted.
3	Awake, responsive to commands only	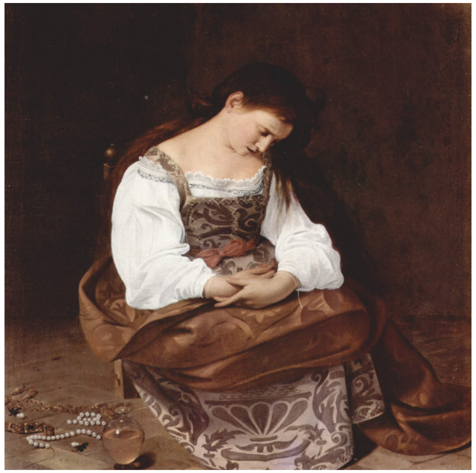	*Penitent Magadalene. Doria Pamphilj Gallery, Rome*<br>The artist painted Mary Magdalene in Penitence Act. The subject appears asleep, with the eyes closed, uninterested in the surronding scene. No emotions are shown through the face. The posture on the seat and the hands held on her lap betray a still active awareness with a postural control.

4	Asleep, brisk response to stimuli light and noise	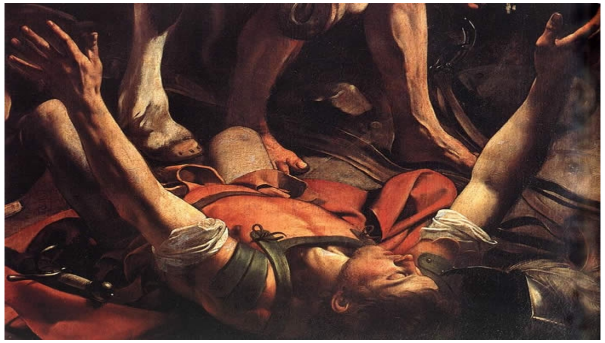	*Conversione di San Paolo (detail), Church of Santa Maria del Popolo, Rome*<br>The painting depicts St. Paul fell down of his horse, after being blinded by the divine light meanwhile he was going to Damascus seeking the authorization to arrest Christians.<br>On the ground, hit by an epiphany, he hears the voice of Jesus, exhorting him to conversion.<br>The subject is lying on the ground, his face expressionless with eyes closed. It seems to be being awakened by the sensory stimulation at the moment.
5	Asleep, response to pain (glabellar tap)	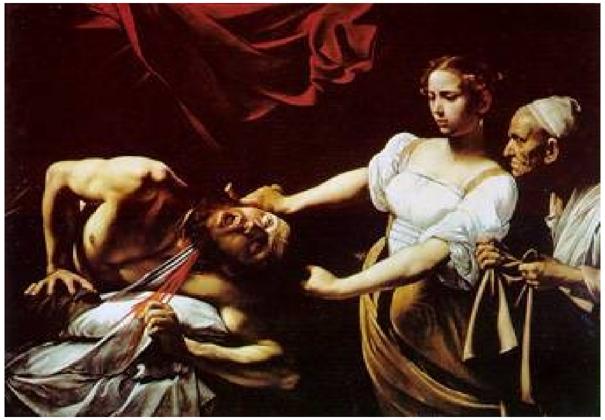	*Giuditta e Oloferne, Galleria Nazionale d'Arte Antica at Palazzo Barberini, Rome*<br>It is depicted the Biblical episode of the beheading of the Assyrian commander Holofernes by Judith, a Jewish widow. The painting is thought to have been inspired by a contemporary news in which a woman, her stepmother and brother, killed her father, after having fallen him asleep with opium. Holofernes caught asleep, is portrayed as visbly contract by grief, the mouth opened in a grimace, wide eyes and tense muscles.

6	Asleep, no response to any stimuli	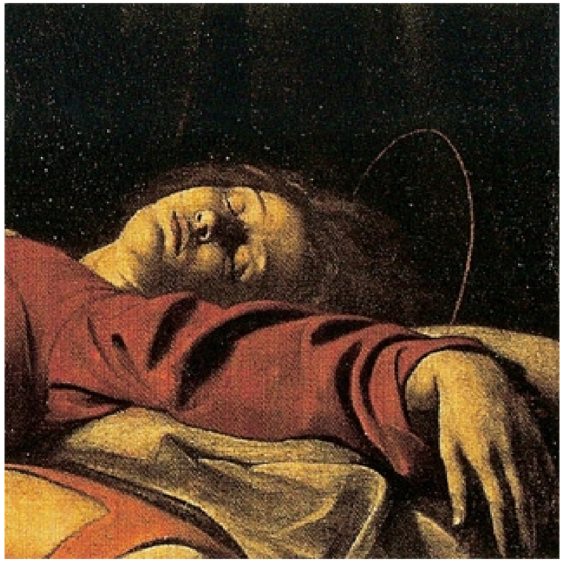	*Morte della vergine (detail), Musée du Louvre, Paris* Madonna is portrayed as dead, while the weeping throng of Apostles crowds around her. The subject seems to be asleep, the body lying on the bed, his open hand hanging beyond the edge, no emotions are conveyed by her face.
